# Triplet Therapy with PD-1 Blockade, Histone Deacetylase Inhibitor, and DNA Methyltransferase Inhibitor Achieves Radiological Response in Refractory Double-Expressor Diffuse Large B-cell Lymphoma with 17p Deletion

**DOI:** 10.1155/2020/8879448

**Published:** 2020-08-26

**Authors:** Runhui Zheng, Xiaobo Chen, Chunyan Wang, Pengfei Qin, Huo Tan, Xiaodan Luo

**Affiliations:** ^1^Department of Hematology, First Affiliated Hospital, Guangzhou Medical University, Guangzhou 510230, China; ^2^Guangzhou Institute of Respiratory Health, Guangzhou Medical University, Guangzhou 510120, China

## Abstract

Double-expressor diffuse large B-cell lymphoma (DLBCL) with *17p* deletion is an aggressive and refractory disease. Immune checkpoint blockade and epigenetic drugs have been widely used, but the efficacy of different combined applications varied. We report a case with “double-expressor” DLBCL treated with a combined regimen which consisted of programmed cell death protein 1 (PD-1) inhibitor, DNA methyltransferase inhibitor (DNMTi), and histone deacetylase inhibitor (HDACi). A 50-year-old man presented with a 6-month history of hoarseness, and 10 days of progressive shortness of breath was diagnosed of DLBCL, stage IV. The patient failed to respond to the 1^st^ line (R-EPOCH: rituximab, etoposide, vincristine, cyclophosphamide, doxorubicin, and dexamethasone), 2^nd^ line (R-EPOCH + lenalidomide + ibrutinib), and a 3^rd^ line chemotherapy combined with PD-1 inhibitor (sintilimab), decitabine, and GDP (gemcitabine, DDP, and dexamethasone). Surprisingly, patient's condition was improved after treatment with PD-1 inhibitor in combination with DNMTi/HDACi. Restaging PET revealed dramatically radiological response.

## 1. Background

Diffuse large B-cell lymphoma (DLBCL) is the most common type of non-Hodgkin lymphoma (NHL) [1, 2]. Approximately 60% of DLBCL patients achieve complete remission (CR) with standard R‐CHOP (rituximab with cyclophosphamide, doxorubicin, vincristine, and prednisolone) chemotherapy [3]. However, 30–40% are refractory to therapy or later relapse (R/R cases), especially those with *17p* deletion and/or *TP53* mutation and high-grade B-cell lymphoma with *MYC* and *BCL2* or *BCL6* translocation [2, 4, 5]. Programmed cell death protein 1 (PD-1) is a key immune checkpoint receptor that is frequently expressed on tumor-inﬁltrating T cells in B-cell lymphomas [6]. In recent years, although therapeutic blockade of PD-1/PD-L1 has shown potential clinical activity in several types of NHL, the efficiency in DLBCL was not as satisfactory as in Classical Hodgkin lymphoma (cHL) [2, 4, 7–10]. The diversity of clinical responses to monotherapy with PD-1/PD-L1 inhibitors is in part explained by genetic heterogeneity and the diversity of signal pathways involved in the development of B-NHL [8, 11]. Therefore, a combinational regimen of PD-1/PD-L1 inhibitors with other synergistic drugs is needed. Here, we report a case in which PD-1 inhibitor in combination with DNA methyltransferase inhibitor (DNMTi) and histone deacetylase inhibitor (HDACi) was administered to a patient diagnosed of refractory “double-expressor” DLBCL with *17p* deletion.

## 2. Case Presentation

**Figure 1:**
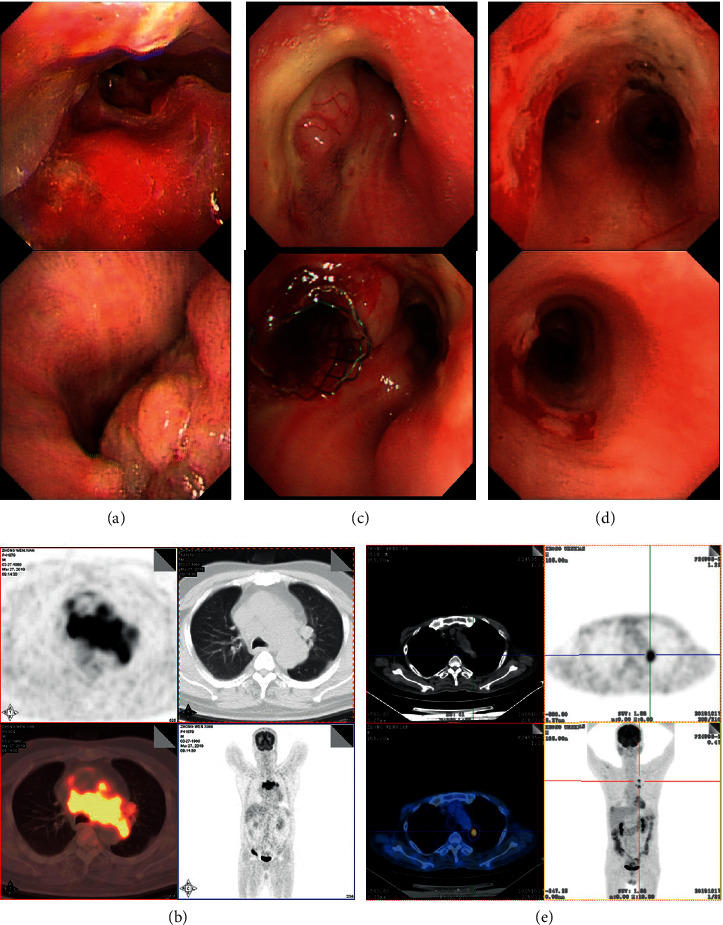
Fiberoptic bronchoscopy and PET/CT images. (a) Fiberoptic bronchoscopy revealed intratracheal mass in right main bronchi with complete right mainstem bronchus occlusion before treatment. (b) PET/CT image showing left hilum and mediastinal mass (41 × 87 × 76 mm, FDG uptake 20.6) with compression of the pulmonary artery, lower tracheal segment, bilateral main bronchi, and superior vena cava. (c) Right mainstem bronchus was completely occluded, and a tracheal stent was implanted. (d) Bronchus occlusion was significantly improved after treatment with PD-1 inhibitor and DNMTi/HDACi. (e) Restaging PET image showing dramatically shrinkage of hilum and mediastinal mass (16 × 15 mm, FDG uptake 8.0) after a triple combination treatment of DNMTi/HDACi plus PD-1 inhibitor.

**Figure 2:**
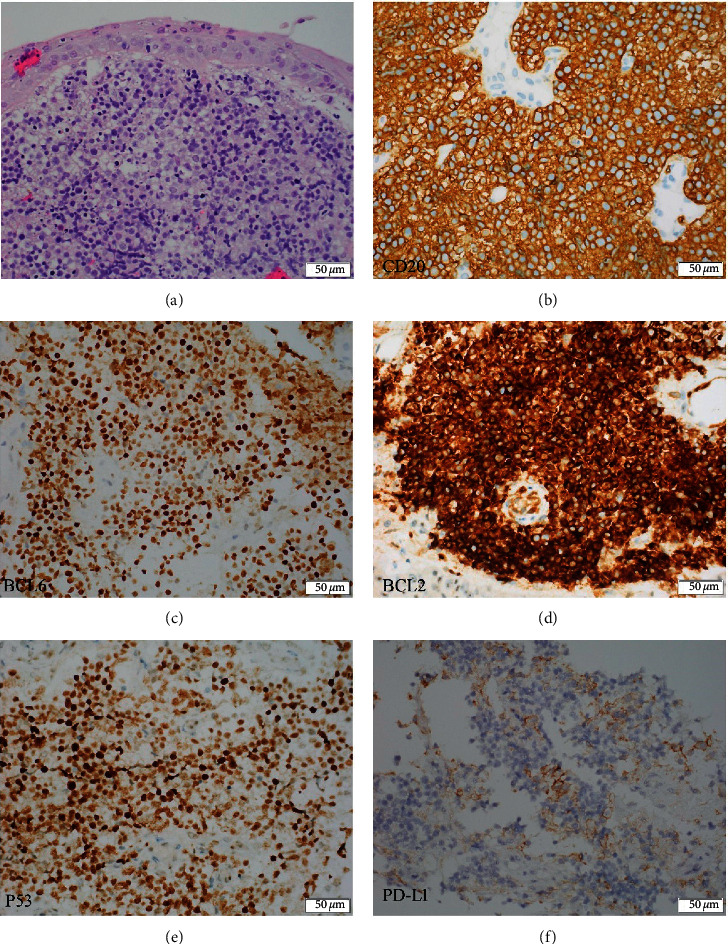
Immunohistochemical characteristics of tumor cells. (a) Transbronchial biopsy of the mediastinal mass (H&E staining) showing a diffuse proliferation of atypical medium- to large-sized lymphoid cells. (b) CD20-positive neoplastic cells from mediastinal biopsy aspirate. (c) BCL6-positive neoplastic cells (70%) from mediastinal biopsy aspirate. (d) BCL2-positive neoplastic cells (95%) from mediastinal biopsy aspirate. (e) P53-positive neoplastic cells from mediastinal biopsy aspirate. (f) PD-L1-positive neoplastic cells from mediastinal biopsy aspirate.

A 50-year-old man presented with a 6-month history of hoarseness and 10 days of progressive shortness of breath. Fiberoptic bronchoscopy revealed intratracheal mass in right main bronchi with complete right mainstem bronchus occlusion (Figure 1(a)). Transbronchial biopsy showed a diffuse proliferation of atypical medium- to large-sized lymphoid cells which were positive for CD20, BCL6 (70%), BCL2 (95%), MUM1 (partial), CD79a, PD-L1 (22C3, 25%), PD-L1 (28–8, 25%), TP53, and c-Myc (40%). They are negative for CD3, CD5, and CD10. The Ki67 proliferative fraction is 80%+ (Figure 2). Fluorescence in situ hybridization (FISH) results demonstrated the presence of *17p* deletion and *BCL-2* rearrangement, while c-Myc translocation was negative. Initial ﬂuorodeoxyglucose positron emission tomography (FDG-PET) revealed left hilum and mediastinal mass (41 × 87 × 76 mm, FDG uptake 20.6) with compression of the pulmonary artery, lower tracheal segment, bilateral main bronchi, and superior vena cava (Figure 1(b)). Vocal cords were involved, and there was a small pericardial effusion. Bone marrow and cerebrospinal fluid are negative for lymphoma. Gene mutation by “Next-generation” sequencing showed *STAT6*, *EZH2*, *TP53*, *KMT2D*, *BCL6*, and *CREBBP* mutation. These findings were consistent with DLBCL, stage IV. The patient was started urgently on dose-adjusted R-EPOCH therapy (rituximab, etoposide, vincristine, cyclophosphamide, doxorubicin, and dexamethasone) since DLBCL patients with poor prognosis treated with R-CHOP usually had shorter remission according to our clinical observations. It is also reported that DA-EPOCH-R produced durable remission in patients with aggressive B-cell lymphomas [12, 13], so we did not risk choosing R-CHOP. A chest computed tomography (CT) scan showed no sign of improvement. In a phase II study from MD Anderson Cancer Center of using rituximab, lenalidomide, and ibrutinib lead in prior to combination with chemotherapy for patients with newly diagnosed DLBCL, the CR rate was 96%. The regimen also has promising activity in R/R Non-Germinal Center B-cell-like DLBCL [14]. Therefore, R2-CHOP-I therapy (rituximab, lenalidomide, ibrutinib, vincristine, cyclophosphamide, doxorubicin, and dexamethasone) was administered as 2^nd^ line. However, the patient developed respiratory distress soon, and a tracheal stent was implanted into the completely occluded bronchus (Figure 1(c)). A repeat PET showed progressive disease. In view of the patient's clinical status with rapidly progressing respiratory distress, a combination regimen with PD-1 inhibitor (Sintilimab, 10 ml: 100 mg), DNMTi (Decitabine, 10 mg), and GDP (gemcitabine, DDP and dexamethasone) was given as 3^rd^ line. Follow-up CT scan showed only a slight improvement (much less than 50%) of bronchus occlusion without the shrinkage of the hilum and mediastinal mass.

For 4^th^ line, a triple combination of decitabine, sintilimab plus HDACi (Chidamide, 5 mg) were administered (decitabine 10 mg i. v. d1-5, Sintilimab 200 mg, i. v., d1, Chidamide 20 mg p. o. twice a week). Chidamide, which inhibits class I HDACs 1, 2, 3, as well as class II HDAC 10, is the first oral subtype-selective histone deacetylase inhibitor approved in China. It was rapidly absorbed after oral administration and exhibited an elimination half-life in plasma of 17–18 hrs. The recommended dose for lymphomas was 10 mg twice per week for 4 consecutive weeks in a 6-week cycle [15, 16]. Right after the 4^th^ line treatment, a signiﬁcant clinical improvement was noted. Bilateral bronchus was clear, and restaging PET 2 weeks later revealed over 90% shrinkage of hilum and mediastinal mass (16 × 15 mm, FDG uptake 8.0, PET-CT score 5) and significant improvement of bronchus occlusion (Figures 1(d) and 1(e)). The patient achieved partial remission (PR), and a total of 3 cycles of this combination therapy were given. The patient had only myelosuppression presented with absolute neutrophil count <1.5 × 10^9^/L and platelets <80 × 10^9^/L which lasted for less than a week. The PR duration was about 40 days until a repeat PET-CT showed PD (41 × 33 mm, FDG uptake 18.4, PET-CT score 5). Then, venetoclax was used due to BCL-2 rearrangement, in combination with Chidamide, Sintilimab, and radiotherapy (50 Gy). Decrease in lesion size and FDG uptake were observed (15 × 13 mm, FDG uptake 1.9, PET-CT score 4). However, PET-CT revealed new metastatic foci (10 × 10 mm, FDG uptake 3.4, PET-CT score 5) on the right cardiodiaphragmatic angle 1 month after radiotherapy. Fortunately, the patient had a twin brother as transplant donor and syngeneic stem cell transplantation was performed about 6 months after diagnosis and CR was confirmed by a repeat PET-CT +35 d posttransplant. The patient is alive without lymphoma, and thymosin alpha-1 and recombinant human interleukin-2 are used to induce GVL. Treatment timescales are shown in Table 1, and response assessment was made according to the Lugano classification [17].

## 3. Discussion

Immune checkpoint blockade has been considered an important breakthrough in cancer treatment. The ﬁeld of clinical trials in DLBCL associated with immune checkpoint blockade such as PD-1/PD-L1 inhibitor is being actively studied. Unlike cHL, the clinical response to monotherapy with PD-1/PD-L1 blockade in R/R DLBCL was not confirmed [2, 4, 7–9]. Results in a phase I trial of nivolumab monotherapy were promising with a response rate of 36% in R/R DLBCL. Clinical trials of different PD-1/PD-L1 inhibitors are ongoing, and a subset of patients experienced progressive disease after a short response [18]. Therefore, there is an urgent need to designate an effective PD-1/PD-L1 inhibitor-based combination regimen for R/R DLBCL.

This “double-expressor” DLBCL with *17p* deletion failed the 1^st^ line chemotherapy and presented with increase in overall tumor burden both in lesion size and FDG uptake after 2^nd^ line chemotherapy. It is difficult to identify whether these responses were “tumor flare” or true disease progression since lenalidomide and ibrutinib were used in the 2^nd^ line treatment. “Tumor flare” has been observed in patients treated with immunomodulatory drugs [19], but aggressive lymphoma is different from solid tumor or chronic lymphocytic leukemia possibly presented with self-limited lymphocytosis or increase in the size of lymph nodes. Increased FDG uptake with a concomitant increase in lesion size was consistent with what was found by fiberoptic bronchoscopy in this case. Thus, the Lugano classification was used to assess lymphoma response rather than the lymphoma response to immunomodulatory therapy criteria (LYRIC) [19]. The most important reason to change the regimen was that the rapid progression of mass could result in suffocating and death. The PD-1 inhibitor was chosen since PD-L1 was positive in 25% of tumor cells. Rapid progression of disease did not allow us to choose monotherapy of PD-1 inhibitor because of the low response rates and lack of durability. On one hand, it is reported that PD-L1 expression on tumor cells in DLBCL was shown to be associated with poor prognosis in DLBCL in most clinical data [7, 20]. One the other hand, resistant DLBCL has been identified to simultaneously utilize multiple pathways that could compensate for the one provided by PD1 blockade. The PD-1/PD-L1 signal pathway turns down the activation of the immune response, and PD-1 blockade only modestly improved T cells proliferation and cytokine production but was insufﬁcient to restore the antitumor activity [21]. By targeting multiple pathways, which in addition to PD1, combination regimen may overcome the resistance to anti-PD1 therapy.

Multiple components of DLBCL microenvironment are affected by epigenetic regulators such as HDACis or DNMTi [22]. In this report, *STAT6, EZH2, TP53, KMT2D, BCL6,* and *CREBBP* mutations are mostly associated with epigenetic dysregulation. After decitabine was added to the treatment, the patient showed a slight improvement rather than progression for the first time. Moreover, mutation of *CREBBP* encoding proteins with established roles in histone acetylation often results in impaired *p53* activation while also promoting the oncogenic effects of *BCL-6* [23]. HDACis as epigenetic modulators could reduce PD-L1 and PD-L2 expression rapidly on tumor cells, upregulate the immune response, and alter the drug resistance [22, 24]. Therefore, a triple combination of DNMTi/HDACi plus PD-1 inhibitor was performed, and the therapy led to a significant radiological response. *EZH2* mutation was also found in this case. It is reported that the EZH2 inhibitor demonstrated an enhanced clinical activity in DLBCL, so EZH2 inhibition could be a promising strategy [25].

Despite this triplet therapy provided very good partial metabolic response which lasted for 40 days, the patient had progressive disease inevitably even after radiotherapy was performed. However, over 50% shrinkage of lesion size and decreased FDG uptake were observed after radiotherapy. Though the presence of bulky disease remains a significant predictor of disease recurrence, radiotherapy as bridging strategy for stem cell transplantation played an important role in reducing tumor burden.

## 4. Conclusions

The combination of epigenetic-modulating agents with immune checkpoint blockade provides exciting avenue for future research. This case suggests that PD-1 blockade in combination with DNMTi/HDACi can be encouraging in refractory DLBCL. Further research is warranted to evaluate this novel therapeutic regimen.

## Figures and Tables

**Table 1:** Treatment timescales.

	Start time	Therapy	Number of cycles	Time for repeat CT/PET	Effect
1^st^	2019/7/19	R-EPOCH: rituximab, etoposide, vincristine, cyclophosphamide, doxorubicin, and dexamethasone	1	2019/8/8 (CT)	Stable disease
2^nd^	2019/8/15	R-EPOCH + lenalidomide (25 mg d1-7) + ibrutinib (420 mg once a day)	1	2019/9/4 (PET)	Progressive metabolic disease
3^rd^	2019/9/5	GDP (gemcitabine, DDP, and dexamethasone) + Sintilimab (200 mg d1) + Decitabine (10 mg d1-5)	1	2019/9/17 (CT)	Stable disease
4^th^	2019/9/29	Sintilimab (200 mg, iv. d1) + Decitabine (10 mg, iv, d1-5) + Chidamide (20 mg, po, twice a week)	3.	2019/10/17 (PET)	Partial metabolic response and >90% shrinkage of mass
	2019/10/22				
	2019/11/12			2019/11/26 (PET)	Progressive metabolic disease
5^th^	2019/11/29	Radiotherapy (50 Gy) + Sintilimab (200 mg d1) + Chidamide (20 mg twice a week) + venetoclax	1	2020-2-5 (PET)	Progressive metabolic disease
	2020/2/10	Syngeneic stem cell transplantation		2020-3-25 (PET)	Complete metabolic response

CT, computed tomography; PET, positron emission tomography. Response assessment was made according to the Lugano classification [17]: Stable disease: <50% decrease from baseline in SPD of up to 6 dominant, measurable nodes, and extranodal sites; no criteria for progressive disease are met. Progressive metabolic disease: PET-CT Score 4 or 5 with an increase in intensity of uptake from baseline and/or new foci consistent with lymphoma at interim or end-of-treatment assessment. Partial metabolic response: PET-CT score 4 or 5 with reduced uptake compared with baseline and residual mass(es) of any size. Complete metabolic response: score 1, 2, or 3 with or without a residual mass on 5 PET-CT score.

## Abbreviations

CR: Complete remission
cHL: Classical Hodgkin lymphoma
DLBCL: Diffuse large B-cell lymphoma
DNMTi: DNA methyltransferase inhibitor
FISH: Fluorescence in situ hybridization
GDP: Gemcitabine, DDP, and dexamethasone
HDACi: Histone deacetylase inhibitor
PD-1: Programmed cell death protein 1
PET: Positron emission tomography
PMBCL: Primary mediastinal B-cell lymphoma
NHL: Non-Hodgkin lymphoma
R‐CHOP: Rituximab with cyclophosphamide, doxorubicin, vincristine, and prednisolone
R-EPOCH: Rituximab, etoposide, vincristine, cyclophosphamide, doxorubicin, and dexamethasone
R/R: Relapse or refractory.
